# Use of a Preliminary Artificial Intelligence-Based Laryngeal Cancer Screening Framework for Low-Resource Settings: Development and Validation Study

**DOI:** 10.2196/66110

**Published:** 2025-10-07

**Authors:** Shao Wei Sean Lam, Min Hun Lee, Michael Dorosan, Samuel Altonji, Hiang Khoon Tan, Walter T Lee

**Affiliations:** 1 Health Services Research Centre, Singapore Health Services Pte Ltd, Ngee Ann Kongsi Discovery Tower Level 6, 20 College Road, Singapore, 169856, Singapore, 65 65767140; 2 School of Computing and Information Systems, Singapore Management University, Singapore, Singapore; 3 Department of Head and Neck Surgery & Communication Sciences, Duke University Health System, Durham, NC, United States; 4 Division of Surgery and Surgical Oncology, Singapore General Hospital and National Cancer Centre Singapore, Singapore, Singapore; 5 SingHealth Duke-NUS Global Health Institute, Duke-National University of Singapore, Singapore, Singapore

**Keywords:** head and neck cancers, flexible nasopharyngoscopy, efficient neural nets, deep learning, cancer triage, machine learning, artificial intelligence

## Abstract

**Background:**

Early-stage diagnosis of laryngeal cancer significantly improves patient survival and quality of life. However, the scarcity of specialists in low-resource settings hinders the timely review of flexible nasopharyngoscopy (FNS) videos, which are essential for accurate triage of at-risk patients.

**Objective:**

We introduce a preliminary AI-based screening framework to address this challenge for the triaging of at-risk patients in low-resource settings. This formative research addresses multiple challenges common in high-dimensional FNS videos: (1) selecting clear, informative images; (2) deriving regions within frames that show an anatomical landmark of interest; and (3) classifying patients into referral grades based on the FNS video frames.

**Methods:**

The system includes an image quality model (IQM) to identify high-quality endoscopic images, which are then fed into a disease classification model (DCM) trained on efficient convolutional neural network (CNN) modules. To validate our approach, we curated a real-world dataset comprising 132 patients from an academic tertiary care center in the United States.

**Results:**

Based on this dataset, we demonstrated that the IQM quality frame selection achieved an area under the receiver operating characteristic curve (AUROC) of 0.895 and an area under the precision-recall curve (AUPRC) of 0.878. When using all the image frames selected by the IQM, the DCM improved its performance by 38% considering the AUROC (from 0.60 to 0.83) and 8% considering the AUPRC (from 0.84 to 0.91). Through an ablation study, it was demonstrated that a minimum of 50 good-quality image frames was required to achieve the improvements. Additionally, an efficient CNN model can achieve 2.5-times-faster inference time than ResNet50.

**Conclusions:**

This study demonstrated the feasibility of an AI-based screening framework designed for low-resource settings, showing its capability to triage patients for higher-level care efficiently. This approach promises substantial benefits for health care accessibility and patient outcomes in regions with limited specialist care in outpatient settings. This research provides necessary evidence to continue the development of a fully validated screening system for low-resource settings.

## Introduction

Head and neck cancers (HNCs) are the 6th most common cancer worldwide, with a disproportionate growth in incidence and mortality in low- and middle-income countries (LMICs), particularly the West-Pacific and Southeast Asia regions [1-3]. Among HNCs, laryngeal cancer can be challenging to diagnose, with nonspecific and mild symptoms in the early stages. Early-stage diagnosis of laryngeal cancer is crucial to improve survival and quality of life [4]. Patients presenting with early-stage cancers have a 60%‐90% chance of cure with local therapy, while those with late-stage cancers have a significantly reduced opportunity for remission [5,6]. In addition, patients with advanced cancers have worse quality of life due to their swallowing, verbal communication, and breathing dysfunctions [7].

The early detection of laryngeal cancer requires highly trained health care providers (eg, otolaryngologists) to visualize and interpret the relevant anatomical structures to detect anomalies. In addition, a definitive diagnosis requires downstream histopathological confirmation. Sophisticated endoscopic equipment, such as flexible nasopharyngoscopy (FNS), is necessary to examine the upper aerodigestive tract for abnormalities [8]. Experts who can perform this examination and interpret the endoscopic videos are limited in many low- and middle-income countries and in low-resource settings [9]. Limited access to specialty care in low- and middle-income countries is apparent for HNCs, with one study estimating the otolaryngologists-to-population ratio in some Asian countries to be as low as 1 per 2,146,000 [10]. This results in missed opportunities for early-stage diagnosis [2]. Technological advancements, particularly in fiberoptic flexible endoscopy and laser systems, have enabled the shift of many laryngological procedures from the operating room to outpatient clinics [11]. In high-volume outpatient settings, trained non-specialists may benefit from artificial intelligence (AI)-based clinical decision support systems encapsulating domain expert knowledge. Clinical decision support systems (CDSS) with embedded clinical practice guidelines, rules, and specialist knowledge may more effectively assess and triage the endoscopies performed by non-specialist health care workers while having the advantage of portability and accessibility [12].

AI, specifically machine learning and deep learning, is increasingly used to detect abnormalities in medical images and support cancer clinical decision-making, including screening, diagnosis, and prognosis [13-16]. The early application of deep learning and machine learning models in laryngeal cancer management has demonstrated the potential for detection capabilities comparable to human experts [16,17]. Deep convolutional neural networks (DCNNs) have been reported to deal with various data modalities for different use cases across the entire care chain [17,18]. These include real-time lesion detection [19-21] and segmentation [19,21], as well as screening, diagnosis [22], management, and prognosis of laryngeal cancers [16].

Deploying these AI models in the imaging practice presents several challenges. Imaging modalities use high-dimensional data. Applications that process single image frames account for the frames’ pixel resolution and other features (eg, color channels). This “curse of dimensionality” effect, confronted by computational AI models, is compounded when considering video streams that capture many sequential image frames. For FNS procedures, the frame count ranges from hundreds to thousands, depending on the frame capture rate and the procedure’s duration.

Furthermore, the high computational requirements of performant AI-based models must be considered in low-resource settings [23-28]. Another concern, especially for video-based procedures, such as screening for laryngeal cancer through FNS, is that the frames of interest may only lie within a range of non-blurry, contiguous frames that capture the anatomical landmark of interest (ie, the region of interest within each frame). This adds the challenge of localizing decision-making to a few clear and relevant regions and frames that best inform case escalation to more advanced diagnostic and treatment procedures. A previous study has proposed manually filtering frames to exclude low-quality frames (ie, blurry, noisy) before making an assessment [17]. Others have suggested various preprocessing steps to improve the quality of input images [29-32]; for instance, Huang et al [31] suggesting using the grayscale adaptive entropy value for setting the threshold to eliminate unclear images and recognize vocal fold disorders.

This formative study introduces an AI-based framework that denoises high-dimensional FNS videos, selects relevant frames, and suggests care escalation decisions through a referral grade classification task. To handle noisy real-world data and select relevant frames, our framework proposes an image quality module (IQM) that conducts a two-step procedure of filtering redundant images using a histogram of gradient-based threshold model and selecting good quality frames using supervised DCNN models. This IQM is used in conjunction with a disease classification module (DCM) that outputs a probability that a case should be escalated to appropriate downstream test and treat procedures. We aim to explore the use of efficient DCNN models and validate whether the proposed framework enhances the performance of correctly classifying cases to appropriate referral grades to address the resource constraints envisioned in less well-resourced settings [26,33].

## Methods

### Data Acquisition

Our study dataset has 132 full-color FNS videos of varying lengths collected from laryngoscopy procedures conducted in the Duke University Health System from December 2019 to December 2020. The shortest video was 5 seconds, while the longest was 165 seconds. The video clips were captured with various orientations, movements, and variable lighting and contrast conditions during the procedure. Some of these patients were healthy (no laryngeal pathology), some had benign disease processes, and some had laryngeal cancer. Patients were excluded if the videos were taken post-laryngectomy or if the larynx was not visualized on the video. Expert clinicians annotated the videos with medical conditions and referral levels for training classification models. The medical conditions were classified into three referral levels by a panel of 4 clinicians (two senior and two junior specialists): Grade 1, no referral required; Grade 2, non-urgent referral or close follow-up in 3‐4 weeks; and Grade 3, urgent referral.

### Ethical Considerations

All FNS videos were de-identified before analysis to protect patient privacy and confidentiality. The study was approved by the Duke University Health System Institutional Review Board (No. Pro00106209). The IRB granted a waiver of informed consent, as the study involved only de-identified data and posed no risk to participants. No compensation was provided to participants. No identifiable individuals appear in any images or materials included in the manuscript or supplementary files.

### AI-Based Framework for Laryngeal Cancer Screening

#### Overview

**Figure 1.**
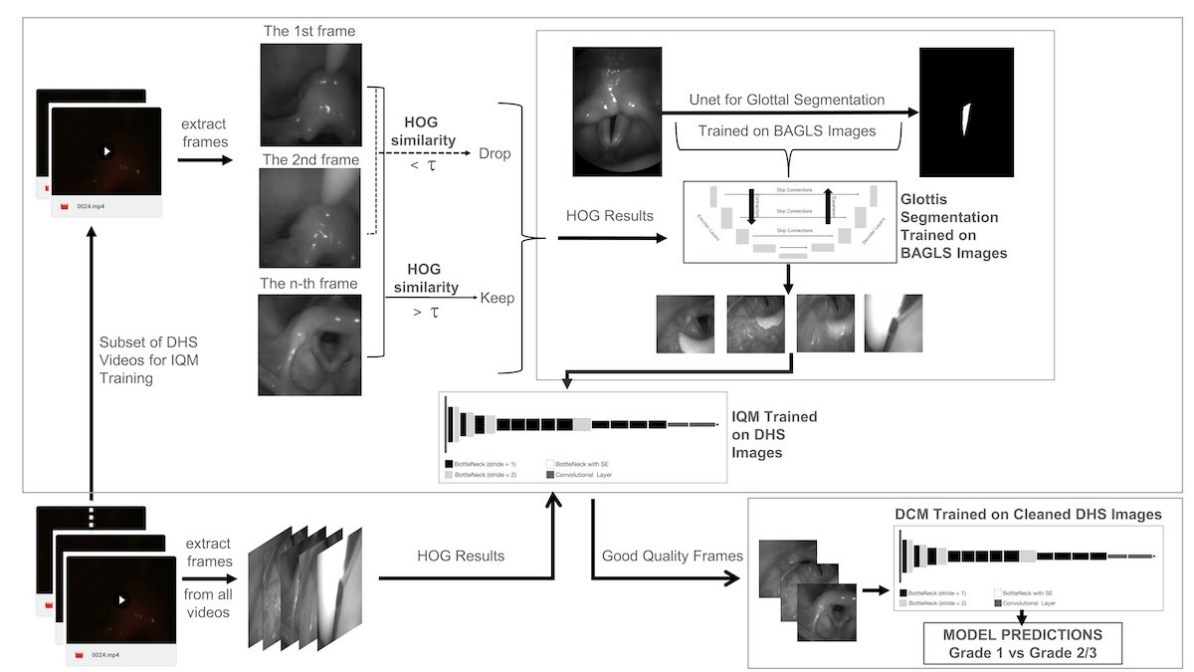
Schematic of the proposed AI-based framework based on the IQM and DCM. AI: artificial intelligence; BAGLS: Benchmark for Automatic Glottis Segmentation; DCM: disease classification module; DHS: Duke University Health System; HOG: histogram of gradients; IQM: image quality module.

Figure 1 shows the proposed framework for screening patients receiving an FNS procedure. The framework includes two main components: the IQM and the DCM. The IQM filters low-quality and irrelevant images through a histogram of gradients-based threshold compared to an indexed 1st frame of each video. A U-Net model, trained with segmentation masks derived from the open-source benchmark for automatic glottis segmentation (BAGLS) dataset [30], was used to generate a labeled dataset for training the IQM to select relevant frames. The trained IQM network is then used to further refine the set of high-quality frames. Using selected frames, we train an efficient AI-based DCM to classify the referral grade. Figure 1 shows the schematic of the training and inference process based on the IQM and DCM.

#### Image Quality Module

We developed a two-stage IQM based on (1) histogram of gradients-based similarity filtering, followed by (2) a U-Net-based DCNN module to identify a set of relevant, good-quality images. The correlation (ie, by cosine similarity) of features from a histogram of gradients [34] was used to evaluate the similarity of contiguous frames with the indexed frame (which is the first frame of relevance in the FNS process). After similarity filtering, the U-Net model was trained to identify the glottal region using the open-source BAGLS dataset [30]. Good-quality images are frames where the glottal area is entirely visible, regardless of the image’s position and the glottis’s size. We then assigned positive and negative quality labels to the Duke University Health System dataset using the U-Net model’s predictions. Negative labels indicate poor quality due to obscuration and blurring by natural bodily secretions and movements or irrelevance, that is, frames not of the glottal region.

#### Disease Classification Module

The 132 unique patient videos were filtered into “good” and “poor” quality frames with the IQM. Sixteen videos were excluded due to insufficient good-quality image frames. The remaining 116 videos were used to train the DCM model using an 80‐20 patient-level train-test split to avoid data leakage. The DCM classifies patients into binary referral grades: non-referral (Grade 1) versus referral (Grades 2 to 3).

To develop the DCM, we compared a baseline CNN model [35], a ResNet50-based model [36], a MobileNetV2-based model [33], and a GhostNet-based model [26] across six validation metrics. The baseline CNN model has six convolutional layers with maximum pooling and batch normalization, adding dropout [37] to the last two layers. ResNet50 employs deep residual learning with skip connections, enabling training of very deep neural networks without the challenge of vanishing gradients [38]. MobileNetV2 employs inverted residuals and depthwise separable convolutions for more efficient performance, intended for mobile and embedded devices. Lastly, GhostNet further enhances the efficiency of computations by generating more feature maps from efficient operations; this results in an even more efficient DCNN suitable for lightweight applications.

#### Model Evaluation

The classification performance for IQM at the image level and DCM at the video (ie, patient) level was evaluated across six validation metrics. The train-test split was determined at the patient level to avoid data leakage. For the patient-level classification, we utilized the concept of bootstrap aggregation to evaluate the average classification probability of image frames within the same video. The primary metrics that describe the quality of the model predictions are accuracy, weighted F1 score, area under the receiver operating characteristic (AUROC), and area under the precision-recall curve (AUPRC). The secondary metrics that describe the efficiency of the models are the total number of floating-point operations in the order of 10^9^ (GFLOPs) [39] and inference times. These outcome metrics describe the efficiency and effectiveness of the algorithm for model training, validation, and inference [23,26,28]. In addition, to address potential data imbalance that may hinder the classification model’s ability to learn minor class patterns, the final selected model was further evaluated with training sample augmentation, and binary focal cross entropy loss [40]. To assess the impact of the IQM in the overall framework, we performed an ablation study [41] in which the DCM classifier was evaluated both with and without IQM-based preprocessing. Specifically, we trained and tested the DCM using input sequences that had undergone the IQM step. In addition, we systematically varied the number of high-quality frames provided to the DCM to examine the effect of input frame count on the classification performance.

## Results

### Comparison of the BAGLS Dataset and the Study Dataset

Table 1 summarizes the BAGLS and the study dataset. The BAGLS dataset has approximately 60% healthy patients, whereas our dataset has 30% healthy patients. The number of frames derived from the patient-level videos is roughly the same ratio. We used the entire BAGLS cohort, comprising 59,250 frames, to label informative frames. The raw dataset comprised 190,978 images derived from 132 patients.

**Table 1.** Summary of cohorts from the BAGLS^a^ dataset and our dataset.

	BAGLS		Study dataset	
Disorder Status	Cohort size (%)	Patient count (%)	Frame count (%)	Cohort size (%)
Healthy (Grade 1)	35,400 (59.7)	382 (59.7)	49,282 (25.8)	40 (30.3)
Unhealthy (Grade 2/3)	23,850 (40.3)	258 (40.3)	141,696 (74.2)	92 (69.7)
Total	59,250 (100)	640 (100)	190,978 (100)	132 (100)

a BAGLS: Benchmark for Automatic Glottis Segmentation.

### Performance of the Image Quality Module

Table 2 compares the test performance of the baseline CNN model [35], ResNet50-based model [36], and GhostNet-based model [26] for the IQM. Although the ResNet50-based model had the best accuracy of 0.833, the best F1 score of 0.832, and the best AUPRC of 0.957, the GhostNet model had comparable performance with the ResNet50 model and the best AUROC score of 0.895 with the fewest GFLOPs for computation.

The IQM model generated 20,040 good-quality frames from 116 patients in the study dataset cohort for the DCM training and test sets. Of these, 34/116 patients (29.3%) were classified as having Grade 1 disease, while the remainder 82/116 (70.7%) were classified as having Grade 2/3 disease. GhostNet resulted in the highest AUC-ROC and AUPRC, while being the most efficient, that is, the lowest GFLOPs.

**Table 2.** Comparison of the performance of the different deep convolutional neural network architectures used in the image quality module (IQM).

Model	Accuracy	F1 score	AUROC^a^	AUPRC^b^	GFLOPs^c^
Baseline convolutional neural network	0.699	0.673	0.724	0.729	50.0
ResNet50	0.833	0.832	0.746	0.957	245.0
GhostNet	0.829	0.827	0.895	0.878	8.7

a AUROC: area under the receiver operating characteristic curve.

b AUPRC: Area under the precision-recall curve.

c GFLOP: Number of floating-point operations in the order of 109.

### Performance of the Disease Classification Module

The ResNet50 and GhostNet DCM achieved accuracy, optimal F1-scores, and AUPRC exceeding 80% at the video-level classification (Table 3). The ResNet50 model’s inference time was 20.44 s, nearly 2.5 times slower than that of GhostNet (7.95 s per batch). Using an inference batch size of 64, 224-pixel-sized images (ie, height and width), ResNet50 had 245.0 GFLOPs, 40 times more than the GhostNet model with 8.7 GFLOPs.

**Table 3.** Performance comparison of different disease classification module (DCM) classifiers at the patient level.

Model	Accuracy	F1 score	AUROC^a^	AUPRC^b^	Inference time (s)	GFLOPs^c^
Convolutional neural network	0.652	0.624	0.595	0.805	8.09	50.0
ResNet50	0.739	0.697	0.667	0.850	16.71	245.0
MobileNetV2	0.696	0.629	0.611	0.833	8.62	20.3
GhostNet	0.870	0.863	0.833	0.912	7.95	8.7

a AUROC: area under the receiver operating characteristic curve.

b AUPRC: Area under the precision-recall curve.

c GFLOP: Number of floating-point operations in the order of 109.

### Ablation Study

As the video-level prediction is based on bootstrap aggregation or bagging [42], the number of frames available to generate disease predictions (post-IQM) will be sensitive to the number of good-quality frames available per patient.

Table 4 shows the sensitivity of predictive quality across the number of high-quality frames. When using all the image frames selected by the IQM, the DCM improved its performance by 38% considering the AUROC (from 0.60 to 0.83) and 8% considering the AUPRC (from 0.84 to 0.91). Our results showed that 50 good-quality frames per patient video were required to outperform the model’s base case without IQM.

**Table 4.** Ablation study results show GhostNet-based disease classification module performance at varying numbers (n) of good-quality frames per patient selected by the image quality module (IQM).

	Accuracy	F1 score	AUROC^a^	AUPRC^b^
Without IQM				
Original number	0.704	0.633	0.600	0.840
With IQM, n				
10	0.676	0.545	0.500	0.839
30	0.622	0.625	0.698	0.877
50	0.784	0.770	0.710	0.884
No limit^c^	0.870	0.863	0.833	0.912

a AUROC: area under the receiver operating characteristic curve.

b AUPRC: area under the precision-recall curve.

c All frames classified as good quality by the IQM are used.

## Discussion

### Principal Findings

This study showed the feasibility of an efficient AI-based screening framework incorporating an image quality filtering module to select high-quality and relevant image frames from FNS videos. Our ablation study demonstrated that the integration of IQM resulted in higher-quality DCM predictions across all the performance metrics at the patient level. Using a minimum of 50 high-quality frames, the DCM showed better predictive performance across all the metrics compared to the base model, where all the image frames were used without the IQM. Addressing the challenges of selecting informative image frames has been identified as a key impediment in developing laryngeal cancer screening algorithms [17]. Our formative research highlights the IQM’s potential to enhance training and inference through effective frame selection.

We leveraged the efficient GhostNet architecture for our IQM and DCM as an alternative to the more resource-intensive ResNet50 model. GhostNet-based models have demonstrated performance comparable to those using less efficient architectures, such as ResNet50 [9]. In our study, the GhostNet-based DCM produced the best model across the validation metrics. The model achieved an accuracy of 87% and a high AUROC (0.833) and AUPRC (0.912) for classification at the patient level, with the optimal F1-score of 0.863 (Table 3). This level of performance, combined with the model’s efficiency, makes it more suitable for integration into low-cost FNS facilities and screening equipment.

Our dataset, comprising 132 patients with 190,978 frames, is smaller than the dataset in a prior study [21], which trained and validated a segmentation model on data from 557 patients with 3933 frames and tested on two additional datasets. Nonetheless, limited patient datasets are common in this field. A related study [43] evaluated a CNN model on 100 patients with 170 images, while another study [44] used data from just 33 patients with 1320 images to assess machine learning algorithms.

Current state-of-the-art computer vision models use transformer-based AI models to classify images, segment pixels, or localize objects within images [45]. While achieving high performance scores on established benchmarks, these models are computationally costly, with computational workloads exceeding those of the ResNets models [46-48]. Studies that compared traditional DCNNs, like those explored in this study, with transformer-based models highlighted greater computational costs and dependence on large training datasets [45,49,50]. Given these limitations, particularly in the context of deployment in low-resource clinical settings, there remains a strong case for exploring simpler, more efficient architectures. This study focused on efficient DCNNs to develop and validate the AI-based IQM-DCM screening framework for laryngeal cancer, emphasizing practical feasibility and predictive performance.

While our FNS videos reflect a realistic clinical setting, they may not fully represent the constraints of low-resource environments. This study serves as a preliminary step towards demonstrating the feasibility of the AI-based IQM-DCM screening framework. Acknowledging the limitations of our dataset, we are actively expanding data collection efforts with multiple partners to further enhance the framework’s robustness and generalizability across diverse low-resourced clinical contexts [16,51]. Recent developments in efficient transformer network models will also be evaluated further to refine the dual-stage screening framework [52,53]. Cost-effectiveness analysis and implementation studies will also be conducted to achieve the envisioned system, which can support referral decisions in low-resource settings [16].

### Conclusion

This study demonstrates the potential of the IQM-DCM framework to be embedded in an AI-based system to support early screening and triaging of patients at risk of laryngeal cancer. This preliminary work provides early evidence supporting the feasibility of this approach. Notably, the IQM-DCM framework, leveraged on lightweight neural network architectures, is shown to outperform conventional CNN models across various effectiveness and efficiency metrics. Future work will expand the dataset, incorporate recent advances in efficient network architectures, and validate the framework across more diverse populations to enhance its generalizability and real-world clinical applicability.

## Abbreviations

AI: artificial intelligence
AUPRC: area under precision-recall curve
AUROC: area under the receiver operating characteristic curve
BAGLS: benchmark for automatic glottis segmentation
CNN: convolutional neural network
DCM: disease classification module
DCNN: deep convolutional neural network
FNS: flexible nasopharyngoscopy
GFLOP: number of floating-point operations in the order of 10^9^
HNC: head and neck cancer
IQM: image quality module
